# The Correlation of HK2 Gene Expression with the Occurrence, Immune Cell Infiltration, and Prognosis of Renal Cell Carcinoma

**DOI:** 10.1155/2022/1452861

**Published:** 2022-02-27

**Authors:** Chunhui Liu, Huibing Li, Hua Huang, Pengyi Zheng, Zhijun Li

**Affiliations:** School of Clinical Medicine, Henan University of Science and Technology, The First Affiliated Hospital of Henan University of Science and Technology, China

## Abstract

**Objectives:**

Hexokinase 2 (HK2) is one of the key factors involved in the development of several human cancers. However, its role in immune cell infiltration (ICI) and tumor development in renal cell carcinoma is not yet known. Thus, we aimed to explore its relationship with ICI, overall survival, and prognosis of renal cell carcinoma.

**Methods:**

In this study, RNA-seq data from renal cancer and normal tissues were extracted from TCGA and the relationship between HK2 expression and pathological features of RCC patients was analyzed using the GEPIA and UALCAN databases. Subsequently, Western blot and qRT-PCR were performed to analyze the protein and mRNA expression of HK2 in renal cell carcinoma tissues and cell lines. Lastly, various bioinformatics tools were applied to determine the immune cell infiltration, survival, and developing prediction model.

**Results:**

The analysis of RNA-seq data revealed a high expression of HK2 in renal cell carcinoma; furthermore, Western blot and qRT-PCR also showed high expression of HK2 in renal cancer tissues and cell lines. The high expression of HK2 showed a significant positive correlation with the advanced stage of the tumor, lymph node metastasis, and worst survival in renal carcinoma patients. The high expression of HK2 was further identified as an independent risk factor of RCC patients; it also showed a significant positive immune cell infiltration RCC tumor microenvironment including macrophages, B cells, neutrophils, dendritic cells, and CD8^+^ T cells.

**Conclusion:**

the expression of HK2 is positively correlated with the immune cell infiltration and prognosis of renal cell carcinoma patients, thus playing an important role in renal cancer development.

## 1. Introduction

Renal cell carcinoma (RCC) is the malignancy of the proximal tubular epithelial cells of the kidney, covering up to 90% of the primary renal malignancies [1]. On the other hand, it is ranked as the most commonly occurring cancer worldwide, specifically in China [2], while recently, a rapid increase in the RCC cases has been noticed [3]. Based on the tumor tissue classification, it has been divided into kidney clear cell carcinoma, papillary carcinoma, and chromophobe cell cancer [4]. The reason for the high frequency of renal cancer is a lack of specific clinical manifestations and features at the early stage; thus, most cases (20–30%) at the time of initial diagnosis showed metastasis [5]. Because most renal cancer patients showed resistance to both chemotherapy and radiotherapy, thus, they mainly rely on surgical resection [6]. Despite recent advancements in targeted therapy and immunotherapy, the response rate in metastatic renal cancer is still very poor [7]. Since the pathogenesis of renal cancer is still unclear, it is necessary to find new potential underlying factors involved in the development and progression of RCC. The abnormalities in cellular metabolism are one of the main causes of carcinogenesis [8]; the tumor cells normally take energy aerobically through mitochondrial oxidative phosphorylation of glucose and anaerobically through glycolysis [9]. The hexokinase (HK), a glycolytic enzyme, initiates the glycolysis by phosphorylating glucose to glucose 6-phosphate [10]; it also regulates the other biosynthetic pathways [11]. In most cancer cells, the HK2 was found upregulated; thus, this explains the high rate of metabolism in cancer cells [12]. The upregulation of HK2 in cancers is mainly associated with tumor progression and mortality [13]. Moreover, the high expression of HK2 has also been associated with drug resistance and metastasis in various cancers [14], thus making HK2 a potential anticancer therapeutic target.

Keeping in mind the important role of HK2 in cellular metabolism, we sought to explore its function in immune cell infiltration and prognosis of renal cell carcinoma. We specifically explored the association of HK2 with the tumor stage, survival, immune cell infiltration, and prognosis in patients with renal cell carcinoma.

## 2. Materials and Methods

### 2.1. Patients and Clinical Specimens

A total of 60 RCC patients were recruited between March 2019 and May 2020 in this study, and 60 paired tissue samples were obtained from the School of Clinical Medicine, Henan University of Science and Technology, Luoyang, China. At the time of operative procedures, tissue samples were immediately frozen in liquid nitrogen and stored at a −80°C freezer for further analysis. We set inclusion criteria for the collection of specimens: (1) use of postoperative pathological examination to confirm renal cell carcinoma, (2) acquisition of patients with no treatment, neither radiotherapy nor chemotherapy, and (3) availability for the follow-up data. The clinical features of the patients were shown in Table 1.

### 2.2. Patients' Approval and Ethical Committee

All the patients who participated in this study were told about the scientific investigations, and their written informed consents were obtained. Furthermore, the protocols of the study were approved by the ethical committee of the School of Clinical Medicine, Henan University of Science and Technology, Luoyang, China. All the procedures and experiments were performed according to the principles stated in the Declaration of Helsinki 1964 and its latest amendments.

### 2.3. Reagents and Antibodies

The following reagents and antibodies were used: Direct-zol RNA MiniPrep Kit (Zymo Research, R2050), RIPA buffer (Abcam#ab156034), chemiluminescent substrate (Thermo Scientific™ #34075), anti-*β*-actin antibody (cat#MAB374, Chemicon, Temecula, CA, USA), and anti-HK2 (CST#2106).

### 2.4. Cell Culturing

Human RCC cell lines, OSRC, ACHN, 786-O, and HK-2 were directly purchased from Shanghai Yaji Biotechnology Co. Ltd., Shanghai, China. The growth of all these cell lines was maintained under normal standard conditions in a controlled humidified atmosphere of 5% CO_2_ and 37C DMEM. All the cell lines OSRC, ACHN, 786-O, and HK-2 were cultured in Dulbecco's modified Eagle's medium (DMEM) (Gibco, USA) supplemented with 10% fetal bovine serum (FBS) (Gibco, USA) and 1% penicillin-streptomycin. All the cell lines were routinely checked for mycoplasma.

### 2.5. RNA Isolation and HK2 Expression

The RNA from patients' paired tissue samples was extracted using the Direct-zol RNA MiniPrep Kit (Zymo Research, R2050) according to manufacturer's instructions. The total RNA from RCC cell lines was isolated using TRIzol® reagent (Ambion; USA). Briefly, 2 × 10^6^ cells were lysed with TRIzol® reagent, followed by chloroform: isopropanol protocol. The quantity and quality of RNA were determined using NanoDrop. The RNA preparations with high quality proceeded with downstream protocols. cDNA was prepared from total RNA using random hexamers and oligo (dT) primers by the TaKaRa Biomedical Technology (Beijing) Co. Ltd. kit following the instructions. PCR conditions used to prepare cDNA were 10 mins at 30°C and 30 mins at 42°C followed by inactivation at 95°C for 5 mins. Later, the qRT-PCRs were performed for mRNA expression quantifications. The qPCR cycling conditions were 95°C for 10 min, followed by 40 cycles of 95°C for 15 s and 60°C for 60 s. All the qPCR experiments were conducted in triplicates. The 2 − ΔΔ*Ct* method was used to calculate the relative expression of HK2 and normalized to GAPDH. The list of primers (forward and reverse) used in this study is as follows HK2 forward: 5′-ATGATCGCCTGCTTATTCACG-3′ and reverse: 5′-CGCCTAGAAATCTCCAGAAGGG-3′; GAPDH forward: 5′-GAACGGGAAGCTCACTGG-3′ and reverse: 5′-GCCTGCTTCACCACCTTCT-3′.

### 2.6. Protein Expression of HK2 by Western Blot

The indicated RCC cell lines cultured in 6-well plates were harvested by washing with cold PBS three times followed by extraction of protein lysates in RIPA buffer (Abcam#ab156034). The concentration of proteins was quantified using the BCA method. Next, we added the 2X Protein Laemmli Buffer (Biorad#1610737EDU) to the lysate and boiled it for 10 mins. 50 *μ*g of proteins was loaded onto 8% SDS PAGE. Upon completion of SDS PAGE, the proteins were transferred onto the polyvinylidene difluoride (PVDF) membrane by using blotting paper and wet transfer assembly. Later, we performed Ponceau red staining to visualize the successful transfer of proteins. The membrane was blocked with 5% nonfat dry milk in tris-buffered saline tween-20 (TBS-T) for an hour at room temperature followed by washing with TBS-T for 15 mins (with the 5 min interval of changing the buffer). Next, we incubated the membrane with the primary antibodies (anti-*β*-actin antibody, cat#MAB374, Chemicon, Temecula, CA, USA, and anti-HK2, CST) overnight at 4°C on a shaking rotator. Lastly, after washing with PBS, we incubated membranes with the HRP-conjugated secondary antibodies for an hour at room temperature. Immunoblots were then examined using chemiluminescent substrate (Thermo Scientific™ #34075) and the Thermo Scientific™ machine was used to capture the high-quality images and quantified using ImageJ software (NIH, USA).

### 2.7. HK2 Expression in Pan-Cancer and KIRC

We used GEPIA (Gene Expression Profiling Interactive Analysis, http://gepia.cancer-pku.cn/) and UALCAN (http://ualcan.path.uab.edu/index.html) databases to estimate the expression of HK2 in a variety of tumors including KIRC. The images and bar graphs were extracted from the respective database's online tools and referenced properly. More specifically, we used UALCA for the correlational analysis of HK2 expression and clinicopathological parameters of the RCC patients. We also validated these data of HK2 expression in RCC patients by qRT-PCR described above in the protocols.

### 2.8. Prognosis and Survival Analysis

The HK2 expression was measured by qRT-PCR in RCC patients. The prognostic values of HK2 expression were quantified by Kaplan-Meier (KM) plots with log-rank test, and the statistical significance was set at *P* < 0.05. We also performed the Cox proportional hazard model to estimate the correlation between the HK2 expression and the prognosis of renal cell carcinoma patients. *P* < 0.05 was considered significant.

### 2.9. Immune Infiltration Analysis

The tumor infiltration of immune cells was obtained from the online comprehensive resource database TIMER (http://timer.cistrome.org/). In short, we performed a systematic analysis to find the correlation of HK2 expression and immune cells of RCC patients by using the online tool of the TIMER database.

### 2.10. Statistical Analysis

We used the SPSS 25.0 and GraphPad Prism 8.0 software for statistical analysis and the GraphPad Prism 8.0 software for analysis and mapping. All the measurements and data were expressed as mean ± standard deviation (SD). For comparison purposes, two groups and multiple groups of measuring data comparison using Student's *t*-tests and one-way ANOVA were performed, respectively. Furthermore, the relationships between the mRNA expression levels of HK-2 in the patients with RCC tissue samples and the clinicopathological characteristics of patients with RCC were analyzed through Pearson's chi-squared test. In addition, we used the Kaplan-Meier survival analysis and Cox proportional hazard model to analyze the association between the HK2 expression and the prognosis of renal cell carcinoma patients. *P* < 0.05 was considered significantly different.

## 3. Results

### 3.1. The Hexokinase 2 Shows Higher Levels of Transcriptional Expression in Pan-Cancer

**Figure 1:**
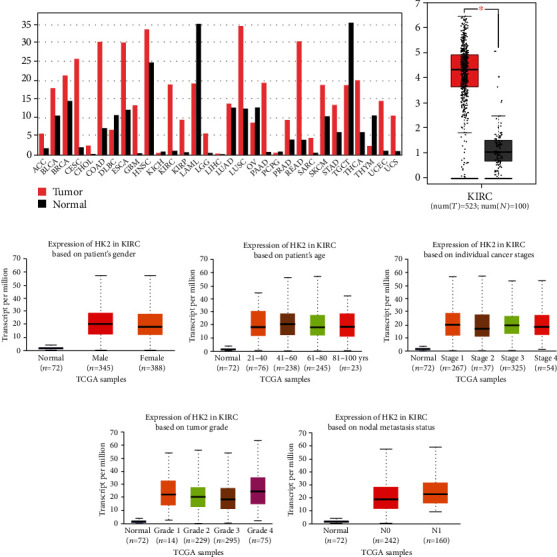
The hexokinase 2 shows higher levels of transcriptional expression in pan-cancer. (a) mRNA expression of HK2 in most of the cancers was higher compared with that of the normal control individuals. (b) KIRC cohort of TCGA and GTEx data to analyze the differential expression of HK2 in tumor and normal tissue samples. (c) Male (*n* = 345) and female (*n* = 188) showed higher expression of HK2. (d) Cancer patients of all ages predominantly expressed higher levels of HK2 compared with normal individuals. (e) From stage 1 to stage 4 of KIRC patients, the expression levels of HK2 were higher compared with those of normal samples. (f) Grading tumors such as grade 1 (well-differentiated low grade) and grade 4 (undifferentiated, high grade), the HK2 expression was observed to be higher in all grades in contrast to normal. (g) For the nodal metastasis status, the expression of HK2 was elevated in N0 and N1 nodes as opposed to that of normal tissues.

Hexokinase (HK) enzymes catalyze the first step of glycolysis. Predominantly, HK has two isoforms HK1 and HK2; the former is widely expressed in adult normal tissues, and the latter is overexpressed in a variety of cancers including renal cell carcinoma [15]. To further understand the role of HK2 in cancer progression, we analyzed the transcriptional expression of HK2 in TCGA-associated cancers using the online GEPIA database. As shown in Figure 1(a), the mRNA expression of HK2 in most of the cancers was higher compared to the normal control individuals (Figure 1(a)). Only a few exceptions were noted in the expression trend of HK2 in analyzed cancers. These include downregulation of HK2 in cancers compared with normal samples, such as LAML, OV, TGCT, and THYM (Figure 1(a)). Controversially, the other studies have suggested higher expression of HK2 in lymphoma tissues [16]; therefore, these discrepancies could be the result of different sample sizes in alternative studies. Next, we utilized the KIRC cohort of TCGA and GTEx data to analyze the differential expression of HK2 in tumor and normal tissue samples. The expression data of 523 KIRC tumors and 100 normal samples showed that HK2 had significantly higher expression in tumor samples compared with normal tissues (Figure 1(b), *P* value = 0.01). To further dissect the expression patterns of HK2 in KIRC tissues, we took advantage of the UALCAN database to explore the relationships between different parameters. For instance, among the analyzed samples, compared with control (*n* = 72), tumors in both the genders, male (*n* = 345) and female (*n* = 188), showed higher expression of HK2 (Figure 1(c)). Similarly, cancer patients of all ages predominantly expressed higher levels of HK2 compared with normal individuals (Figure 1(d)). Moreover, from stage 1 to stage 4 of KIRC patients, the expression levels of HK2 were opposed higher to normal samples (Figure 1(e)). In addition, when analyzed for grading of tumors such as grade 1 (well-differentiated low grade) and grade 4 (undifferentiated, high grade), the HK2 expression was observed to be higher in all grades in contrast to that of normal ones (Figure 1(f)). Lastly, when checked for the nodal metastasis status, the expression of HK2 was elevated in N0 and N1 nodes as opposed to normal tissues (Figure 1(g)). All of the above expressional data suggests that HK2 is highly expressed in KIRC tissues irrespective of clinicopathological features of the tumor, age, and gender of patients.

### 3.2. Human Renal Cell Carcinoma Tissues and Cell Lines Have Elevated Expression of Hexokinase 2

**Figure 2:**
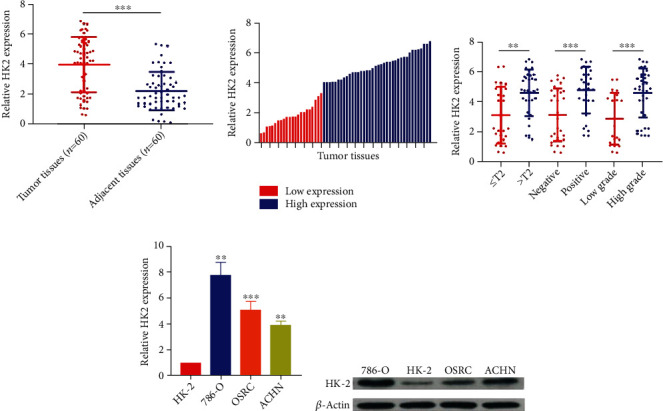
Human renal cell carcinoma tissues and cell lines have elevated expression of hexokinase 2. (a) The expression of HK2 was significantly higher in tumor tissues in contrast with normal tissue samples. (b) The low-expression group has an expression level of HK2 lower than the mean value of ~4, and the high-expression group has higher expression levels than the mean values. (c) High expression of HK2 groups was shown in the T2 tumor stage and undifferentiated high-grade columns, whereas low-expression groups were found in the T1 tumor stage and differentiated low-grade RCC tumors. (d) mRNA and protein expression levels of HK2 in renal cancer and normal cell lines. (e) HK2 protein levels were also elevated in tumor cell lines in contrast with the normal cell line.

To recapitulate the results obtained from TCGA expressional data, we selected 60 renal cell carcinoma (RCC) tissues and three renal cancer cell lines (OSRC, ACHN, and 786-O) along with 60 normal tissues and one renal normal cell line (HK-2). Next, we performed real-time quantitative PCR to determine the expression of HK2 in RCC tumor and normal tissue samples. As expected, we discovered that like pan-cancer results, the expression of HK2 was significantly higher in tumor tissues in contrast to normal tissue samples (Figure 2(a), *P* value < 0.0001). Next, we divided tumors into two expression groups: low-expression group (*n* = 22) and high-expression group (*n* = 38). The low-expression group has an expression level of HK2 lower than the mean value of ~4, and the high-expression group has higher expressions than the mean values (Figure 2(b)). We further utilized these low- and high-expression groups to explore the correlation patterns between HK2 expressions and clinicopathological features of RCC. To this end, we found that the higher expression of HK2 was positively correlated with worse clinicopathological features of RCC (Figure 2(c)). In short, high-expression groups were in the T2 tumor stage and undifferentiated high-grade columns of graphs were shown, whereas low-expression groups were found in the T1 tumor stage and differentiated low-grade RCC tumors (Figure 2(c)). To further add on clinicopathological features, the correlation of HK2 expressions for age and gender was not statistically significant (Table 1). Next, we also determined the mRNA and protein expression levels of HK2 in renal cancer and normal cell lines. For this purpose, we isolated RNA and protein lysates from HK-2 (normal cell line), OSRC, ACHN, and 786-O (renal cancer cell lines) followed by real-time quantitative PCR and Western blot analysis, respectively. Interestingly, we noticed similar expression patterns of HK2 expressions (for both mRNA and protein) as previously found in RCC tissues and normal samples. Briefly, all three RCC cancer cell lines OSRC, ACHN, and 786-O showed higher levels of HK2 mRNA expression when compared with expression in normal cell line HK-2 (Figure 2(d)). Similarly, HK2 protein levels were also elevated in tumor cell lines in contrast with the normal cell line (Figure 2(e)). These results suggest that HK2 expression (at both mRNA and protein levels) was upregulated in RCC tissues.

### 3.3. The Relationship between HK2 Expression and Prognosis RCC Patients

**Figure 3:**
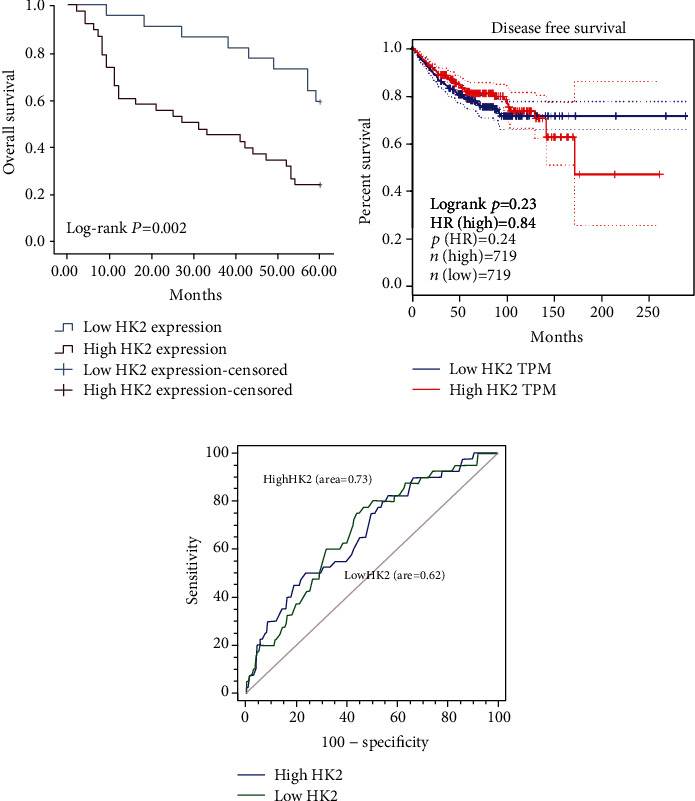
The relationship between HK2 expression and prognosis in RCC patients. (a) Overall survival rates between RCC patients with low expression of HK2 and high expression of HK2. (b) RFS between RCC patients with low expression of HK2 and high expression of HK2. (c) ROC curves between RCC patients with low expression of HK2 (area = 0.62) and high expression of HK2 (area = 0.73).

Above, we have established that HK2 overexpression is positively correlated with the tumor stage, differentiation, and lymph node metastasis, thereby associated with the development of RCC. In previous studies, it has been well known that elevated expression of HK2 has prognostic values in a variety of solid tumors [17]. Therefore, we next explored the prognostic potential of HK2 expression in RCC patients. To find the association of HK2 expression and prognosis of RCC patients, we used Kaplan-Meier survival analysis. Unsurprisingly, the patients with high-expression levels of HK2 had a significantly low overall survival rate than that of patients with low expression of HK2 (Figure 3(a)).

Next, we tried to find the relationship between potentially interacting covariates of HK2 expression with RCC patients using multivariate analysis. To achieve this, we carried out COX proportional risk model analysis. Our results revealed that the expression of HK2, lymph node metastasis, and tumor stage was significantly correlated with the OS rate of RCC patients in univariate analysis (Table 2). However, multivariate analysis showed that expression of HK2 was an independent prognostic risk factor for RCC patients (Table 2). These results highlight the importance of HK2 as a prognostic marker for RCC patients.

### 3.4. HK2 Expression Was Correlated with the Level of Immune Cell Infiltration in RCC Patients

**Figure 4:**
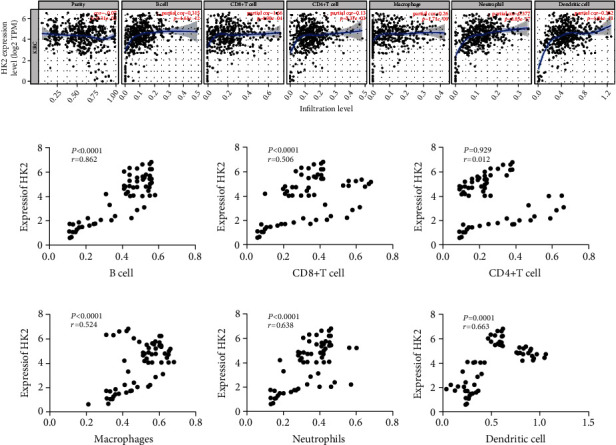
HK2 expression was correlated with the level of immune cell infiltration in RCC patients. (a) The correlation between HK2 expression and immune infiltration cells including B cells, macrophages, neutrophils, dendritic cells, CD8^+^ T, and CD4^+^ T cells. (b–g) Pearson correlation analysis of HK2 expression and immune infiltration cells including B cells, macrophages, neutrophils, dendritic cells, CD8^+^ T, and CD4^+^ T cells.

Tumor heterogeneity could arise from unique compositions of immune cells in tumor microenvironments. Therefore, infiltrating immune cells can significantly contribute to efficacy and patient prognosis [18]. To further explore the prognostic values of HK2 in association with tumor-infiltrating immune cells, we checked six types of invasive immune cells in RCC patients using the TIMER database. Our analysis revealed that the elevated expression of HK2 was positively correlated with immune infiltration cells that includes B cells (*R* = 0.315, *P* = 4.61*E* − 12), macrophages (*R* = 0.28, *P* = 1.71*E* − 09), neutrophils (*R* = 0.377, *P* = 0.85*E* − 17), dendritic cells (*R* = 0.362, *P* = 4.94*E* − 11), CD8^+^ T (*R* = 0.68, *P* = 4.90*E* − 04), and CD4^+^ T cells (*R* = 0.13, *P* = 5.37*E* − 03) (Figure 4(a)). To further verify the correlation status of HK2 expression with immune infiltration cells, we used Pearson correlation analysis. As expected, we discovered that the expression of HK2 in RCC patients was significantly positively correlated with tumor immune-infiltrating cells (Figures 4(b)–4(g)). This correlation was strongly noticed for CD4^+^ T cells with an *R* value of 0.929 (Figure 4(d), *P* = 0.012) and for B cell (*R* = 0.862, *P* = 0.0001) (Figure 4(b)). These results explain the notion that HK2 expression is associated with tumor immune-infiltrating cells in RCC patients.

### 3.5. HK2 Expression Was Correlated with the Level of Immune Marker Genes in RCC Patients

**Figure 5:**
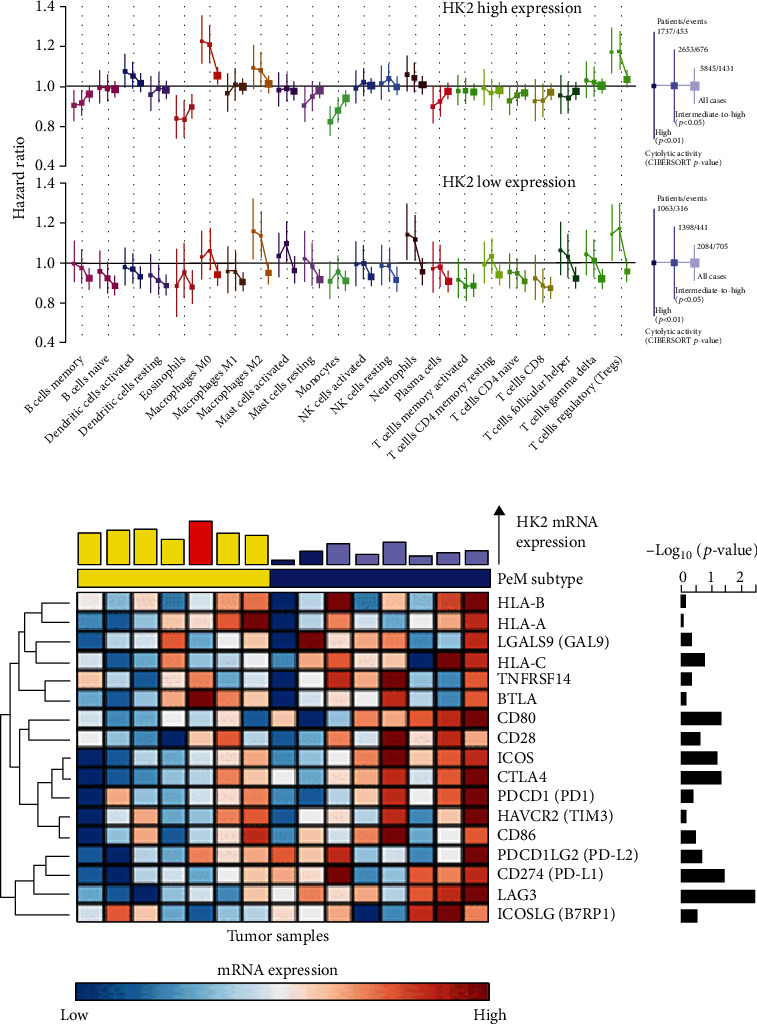
HK2 expression was correlated with the level of immune marker genes in RCC patients. (a) Hazard ratios for RCC patients with high expression of HK2 and low expression of HK2 were defined by the CIBERSORT *P* value. Boxes represent hazard ratios, and vertical lines are 95% confidence intervals. (b) The bar plot of the top of the heat map indicates HK2 mRNA expression levels. The colors on the bar indicate the HK2 copy number status. The bar plot on the right represents the negative log10 of the Wilcoxon signed-rank test *P* value of individual immune checkpoint receptors. The expression levels are log2 transformed and mean normalized.

Significant mRNA expression differences in immune checkpoint receptors LAG3, PD1, CTLA4, CD28, ICOS, BTLA, and HAVCR2 between high expression of HK2 and low expression of HK2 were depicted in RCC patients. Other genes in the figures are interacting receptors of the immune checkpoint markers mentioned above (Figures 5(a) and 5(b)).

## 4. Discussion

Renal cell carcinoma is one of the most common malignancies of the urinary system [19], having a high mortality rate due to the lack of early diagnosis and unavailability of suitable molecular targets [20]. At present, surgical removal along with radiotherapy and chemotherapy is an available option for the treatment of renal cancer [21]. Unfortunately, it has a high recurrence rate even after surgical removal and chemotherapy or radiotherapy and chemotherapy [6]. Even with time, the rate of drug resistance is increasing thus having a high metastasis rate and poor prognosis [22–24]. Overall, the therapeutic outcomes in advanced-stage tumors are not convincing. Therefore, finding new effective diagnostic and therapeutic markers is indispensable.

HK2 catalyzes glycolysis and overexpresses in a variety of tumors [25]; the overexpression of HK2 has been associated with tumor growth and metastasis [26]. In some cancers, the binding ability of HK2 to mitochondria significantly increased which reduces the permeability of the mitochondrial outer membrane, thus enhancing the catalytic activity of HK2 and thus glycolysis [27], ultimately leading to tumor growth and metastasis [28]. The excessive glycolysis rate increases the lactic acid production in the tumor microenvironment which further hinders the antitumor drugs/chemotherapy activity [29]. However, in cancer cells, downstream glycolytic activity reduces the transfer of glucose to branching metabolic pathways [30]. Several studies show that the HK2 is also important for the glucose transfer to these branching pathways in cancer cells [31]. As compared with HK1 and HK3, both N-terminal and C-terminal domains of HK2 have high catalytic activity [32]. However, the acceleration of anabolism in cancer cells requires stronger HK activity; thus, HK2 expresses in the greater amount [33]. However, silencing HK2 expression can suppress proliferation, migration, and invasion of gallbladder cancer cell lines due to significantly reduced glucose consumption and lactate production [33]. In hepatocellular carcinoma, HK2 knockout can inhibit glycolysis promoting oxidative phosphorylation of glucose and increase the sensitivity of cancer cell lines to metformin [34]. In bladder cancer, UMUC-3 cells show high HK2 expression and low HK1 expression; the inhibiting HK2 showed reduced glucose consumption, lactic acid production, and glycolysis [35]. Some inhibitors of HK2, such as 2-deoxyglucose and 3-bromopyruvate, can inhibit glycolysis of a variety of tumor cells and exhibit anticancer effects [36], suggesting that HK2 may be an effective anticancer target.

HK2 has been identified as a potential molecular marker of prognosis in gastric adenocarcinoma, glioblastoma multiforme, laryngeal squamous cell carcinoma, and breast cancer [37–40]. However, few studies have shown the correlation between HK2 expression and prognosis in RCC. In the current study, we first analyzed the GEPIA database and found higher expression of HK2 in most cancers including renal cell carcinoma. Subsequently, the HK2 expression in RCC tissues and adjacent normal tissues was experimentally verified showing similar results with the GEPIA database. The HK2 expression in renal carcinoma cell lines was also significantly higher than that in normal renal cell lines. Furthermore, the HK2 expression was positively correlated with lymph node metastasis, histopathological grade, and tumor stage in renal cell carcinoma patients. Thus, we believe that HK2 could also be used as an independent prognostic factor in RCC patients. Further analyses confirmed the positive correlation of HK2 with immune cell infiltration in renal cell carcinoma tissues.

This may be because the glycolysis-mediated levels of immune cell infiltration and immunotherapy induce resistance in tumors [41]. It has been found that the rate of glycolysis in tumors is connected with the immune resistance of melanoma [42]. On the other hand, in mice with early-stage cancer, bone marrow neutrophils spontaneously migrate to tissues more often due to the increased rate of glycolysis, oxidative phosphorylation, and ATP production [43]. Similarly, we observed high infiltration of neutrophils in the current RCC patients having high HK2 expression. Furthermore, a low number of CD8^+^ T cells were also detected in the current analysis. Previously, proteomic analysis revealed a positive correlation between the reduced infiltration of CD8^+^ T cell and the increase of glycolysis in highly microsatellite unstable tumors [44], which indicated that reducing the glycolysis rate through inhibiting either the HK2 activity or other enzymes may potentially overcome drug resistance and control tumor growth. However, current findings show a significant association of HK2 with immune cell infiltration, survival, and prognosis of renal cancer patients. The current study has some limitations such as HK2 requires further experimental validation in clinical setup as well as in animal models. The specific underlying mechanisms of action of HK2 in immune cell infiltration are yet unknown.

## 5. Conclusion

In conclusion, upregulated HK2 expression promotes glycolysis and participates in the tumorigenesis and progression of RCC, playing an increasingly vital role in renal cancer. Current analyses showed that HK2 is positively associated with the infiltration of immune cells in the RCC tumor microenvironment which is a key factor in tumor development. High expression of HK2 was also associated with the poor survival and prognosis of renal cancer patients. Based on these findings, we conclude that reducing the glycolysis rate via inhibiting HK2 may provide treatment success in RCC.

## Figures and Tables

**Table 1:** The association between the HK2 expression level in RCC and clinicopathological features of renal carcinoma patients (*n* = 60).

Clinicopathological features	HK2 expression			
	Low no. cases	High no. cases	Chi-squared test	*P* value
All patients	(*n* = 22)	(*n* = 38)		
Gender			0.003	0.957
Male	12	21		
Female	10	17		
Age (years)			0.012	0.913
≤55	9	15		
>55	13	23		
Tumor stage			5.831	0.016
≤T2	14	12		
>T2	8	26		
Lymph node metastasis			4.506	0.034
Negative	16	14		
Positive	6	24		
Pathologic grade				
Low grade	12	10	7.177	0.007
High grade	10	28		

**Table 2:** Univariate and multivariate analyses of overall survival in patients with renal cell carcinoma.

Features	Univariate analysis			Multivariate analysis		
	HR	95% CI	*P*	HR	95% CI	*P*
Gender			0.106			
Male vs female	1.726	0.891-3.344				
Ages (years)			0.134			
≤55 vs >55	0.602	0.311-1.169				
Pathologic grade			0.404			
Low grade vs high grade	1.33	0.680-2.601				
Tumor stage			0.03			0.167
≤T2 vs >T2	0.474	0.242-0.930		0.615	0.309-1.226	
Lymph-node metastasis			0.012			0.14
Negative vs positive	0.433	0.225-0.834		0.597	0.301-1.185	
HK2 expression			0.003			0.04
Low vs high	0.32	0.151-0.681		0.431	0.193-0.963	

HR: hazard ratio; CI: confidence interval.

## Data Availability

The data used to support this study is available from the corresponding author upon request.
